# Stress Response Leading to Resistance in Glioblastoma—The Need for Innovative Radiotherapy (iRT) Concepts

**DOI:** 10.3390/cancers8010015

**Published:** 2016-01-13

**Authors:** Stephanie E. Combs, Thomas E. Schmid, Peter Vaupel, Gabriele Multhoff

**Affiliations:** 1 Department of Radiation Oncology, Klinikum rechts der Isar, Technische Universität München, Munich 81675, Germany; t.e.schmid@tum.de (T.E.S.); peter.vaupel@tum.de (P.V.); Gabriele.multhoff@tum.de (G.M.); 2 Institute of Innovative Radiotherapy (iRT), Helmholtz Zentrum München, Neuherberg 85764, Germany

**Keywords:** glioblastoma, immune system, hypoxia, particle therapy, radiation, imaging

## Abstract

Glioblastoma (GBM) is the most common and most aggressive malignant primary brain tumor in adults. In spite of multimodal therapy concepts, consisting of surgery, radiotherapy and chemotherapy, the median survival, merely 15–18 months, is still poor. Mechanisms for resistance of GBM to radio(chemo)therapy are not fully understood yet and due to the genetic heterogeneity within the tumor including radiation-resistant tumor stem cells, there are several factors leading to therapy failure. Recent research revealed that, hypoxia during radiation and miRNAs may adversely affect the therapeutic response to radiotherapy. Further molecular alterations and prognostic markers like the DNA-repair protein O6-methylguanine-DNA methyltransferase (MGMT), anti-apoptotic molecular chaperones, and/or the activity of aldehyde dehydrogenase 1 (ALDH1) have also been identified to play a role in the sensitivity to cytostatic agents. Latest approaches in the field of radiotherapy to use particle irradiation or dose escalation strategies including modern molecular imaging, however, need further evaluation with regard to long-term outcome. In this review we focus on current information about the mechanisms and markers that mediate resistance to radio(chemo)therapy, and discuss the opportunities of Innovative Radiotherapy (iRT) concepts to improve treatment options for GBM patients.

## 1. Introduction

Glioblastoma (GBM, classified by World Health Organization as grade IV astrocytoma), is the most common primary brain tumor in adults. The most important characteristics, which are responsible for the aggressiveness and drug resistance of GBM are high cell proliferation rate, genetic instability, diffuse infiltration and high angiogenesis [1]. Presently the standard of care treatment to improve the clinical outcome of GBM patients is surgery followed by a combination of radiation and adjuvant chemotherapy with temozolomide (TMZ) [2]. Nevertheless the median survival of the patients is only 15–18 months after diagnosis [3]. The recurrence of the GBM, which occurs in nearly all tumor patients is situated in most cases within 2–3 cm of the original resection margin, suggesting that therapeutic resistance is a frequent problem of this tumor [4].

The aim to improve outcome in patients with GBM is the focus of many research groups worldwide: Effort has been put on the increase in resection extent, including microsurgical techniques, imaging measures such as 5-aminolevulinic acid (5-ALA), intraoperative MR-imaging, as well as bold approaches with supramarginal resection; integration of functional imaging identifies eloquent regions and guides the neurosurgeon precisely along side-effect-oriented resection borders [5,6]. It is known that the extent of resection is currently the central prognostic factor: This is beyond dispute in the primary setting, and more and more evidence confirms a central role of resection for recurrent gliomas [7,8].

The necessity of adjuvant treatment is also undisputable: Available measures include systemic treatments (chemotherapy, immunotherapy, molecular-targeted treatments), radiotherapy, vaccinations, as well as other novel gadgets including concepts on magnetic field stimulation. Of all, the central role of radiation therapy has been clearly established over the last centuries. For GBM, a clear dose-response relationship is known; older studies by Walker and colleagues already demonstrated that doses of 60 Gy can significantly increase outcome, and based on these studies the standard dosing scheme of 60 Gy in 2 Gy fractions was established [9,10]. Older techniques were limited by precision, thus exposure of normal tissue was high and the risk for treatment-related side effects increased steeply following the dose-response-relationship for normal brain tissue. Escalation strategies did not show a beneficial risk-benefit profile, and altered fractionation did not convey into a clinical benefit [11,12,13].

### Improvements in Technical Equipment Made Further Dose-Increases Possible

Dose-escalation using a stereotactic boost was approached within an EORTC-trial, with only modest success [14]. The benefit of particle therapy was evaluated with a total dose of 90 Gy equivalent, with high rates of symptomatic necrosis necessitating neurosurgical intervention, however, also with increased overall survival [13]. Main criticism might be the not yet understood real value of the proton relative biological effectiveness (RBE), and the imaging applied within the study as well as target-volume concepts.

Reflecting, however, the unchallenged potency of radiation, Innovative Radiotherapy (iRT) concepts for the treatment of GBM are necessary. Dose escalation to limited regions based on modern imaging might potentially re-vitalize a role for proton therapy in this context. Making use of environmental properties as well as the tumor micromilieu, and taking into account individual radiation sensitivity as well as immunogenic properties of the tumor have significant potential for innovative and highly effective treatments.

## 2. Exploitation of Hypoxia in Human Glioblastomas

Hypoxia (*i.e.*, critically reduced levels of oxygenation) is present in many tumor sites including uterine cervix, head and neck, prostate, breast and soft tissue sarcomas [15]. Systematic studies on the oxygenation status of human tumors have shown that the existence of hypoxic/anoxic subvolumes is a pathophysiological trait in these malignancies. Hypoxic tissue areas show complex spatial and temporal heterogeneities, both within and between tumors of the same entity, and reoxygenation phenomena following phases of intermittent hypoxia. The pathogenesis of tumor hypoxia is multifactorial with contributions from both acute and chronic factors [16]. Despite the long history of chronic and acute hypoxia, their relative importance and fraction in a given tumor remains controversial [17]. Substantial changes in the oxygenation status can occur within the distance of a few cell layers, explaining the inability to adequately assess this crucial trait in the clinical setting. At present, tumor hypoxia is recognized as a principal parameter influencing the disease outcome [18].

For many years, tumor hypoxia has been regarded as an obstacle to the local control of solid tumors treated with standard radiotherapy, some chemotherapies, immunotherapy and photodynamic therapy. During the last two decades, accumulated evidence invariably demonstrates that hypoxia has a strong negative impact driving cancer cells toward a more aggressive phenotype, resulting from an increased mutagenicity (<0.1% O_2_, severe hypoxia), and a hypoxia-driven regulation of a plethora of genes promoting changes of the proteome and metabolome (<1% O_2_, modest-to-moderate hypoxia), ultimately leading to a poorer patient prognosis [19,20,21]. In addition, hypoxia can enhance the expression of stem cell markers (e.g., [22,23]) and can lead to an inhibition of innate and adaptive antitumor immune response [24].

This wide range of hypoxia-associated detrimental effects makes it mandatory to include assessments of the oxygenation status of tumors, gliomas included, in treatment planning, for monitoring individual responses to anti-cancer therapies and, finally, to predict treatment outcome.

Direct measurements of oxygen tensions (pO_2_-values) performed so far on primary brain tumors convincingly show the existence of areas/subvolumes of severe hypoxia in both low- and high-grade gliomas [15,25,26,27,28,29,30,31,32,33,34,35]. As was also seen in other tumor types characterized so far [15], pO_2_-values varied widely among patients [33] and were distinctly lower than in the normal brain tissue [26,27]. An association between oxygenation status and clinical tumor size has so far not been demonstrated. There is evidence, that the extent of hypoxia may be grade-dependent [29,30,35] with high-grade gliomas exhibiting a poorer oxygenation status than low-grade tumors. The latter finding has been substantiated using binding of the exogenous hypoxia marker EF5, a 2-nitroimidazole agent [34].

By pooling of pretherapeutic pO_2_-data from 73 glioma patients, it has been possible to differentiate between low-grade (median pO_2_ = 15 mmHg) and high-grade gliomas (median pO_2_ = 5 mmHg). On average, the grand median pO_2_ in a total of 104 gliomas was 13 mmHg (range: 3–22 mmHg) [15]. This low median pO_2_ in high-grade gliomas is comparable to values assessed in severely hypoxic cancers of the pancreas and of the bile duct [15,36].

In order to further characterize the oxygenation status of glioblastomas multiformes (GBM), hypoxic fractions (HFs), *i.e.*, the relative frequency of hypoxic pO_2_-values measured, have been determined: HF2.5 = 26% (pO_2_ < 2.5 mmHg), HF5 = 46% (pO_2_ < 5 mmHg) and HF10 = 56% (pO_2_ < 10 mmHg) [15]. Mean pO_2_-values obtained in human gliomas are on average distinctly lower than in normal brain (median: approx. 25 mmHg with practically no pO_2_ values < 2.5 mmHg; HF5 = 5%, HF10 = 13%) indicating a physiological (“normal”), well balanced oxygenation status.

Besides using exogenous hypoxia markers (e.g., EF5 [37]), qualitative assessment of hypoxia in human gliomas down to the single cell level is also possible with tissue-based endogenous, hypoxia-related markers (e.g., HIF-1α, CA IX, osteopontin, VEGF, erythropoietin, GLUT-1). A large number of studies has shown that a high expression level of proteins of the HIF-1-mediated transcriptional response reaction—whether they are caused by hypoxia or not—are accompanied by a worsening of the patients’ prognosis, and that these proteins are causally involved in tumor progression and resistance to therapy (e.g., [38,39,40]). Based on the differential expression of endogenous, tissue-based markers, it has been concluded that (a) a combination of hypoxia-related markers is more robust than a single marker for predicting survival of patients with high-grade gliomas, and (b) hypoxia-related genes are strongly expressed particularly in GBM and to a much lesser extent in diffused or anaplastic astrocytomas [38,39,41].

The knowledge of hypoxia and novel identification methods of hypoxic regions, e.g., using PET-imaging, together with radiation therapy might be one of the Innovative Radiotherapy (iRT) approaches potentially moving the risk-benefit profile in favor of increased outcome.

## 3. Targeting of Heat Shock Proteins (HSPs) and Other Related Factors

Making use of the immune system for oncological treatment has moved into focus for several indications. Novel medications have been generated and for certain indications are an essential part in standard treatment, such as malignant melanoma or lung cancer. Several approaches for targeting the immune system are under investigation.

Heat shock proteins (HSPs) are highly conserved molecular chaperones that are classified in different families including HSP27, HSP40, HSP60, HSP70, HSP90 and HSP110 according to their approximate molecular weights. Especially members of the Hsp27, Hsp70 and HSP90α families are frequently found to be upregulated in many tumor types including brain tumors [42] to prevent tumor cells from radiochemotherapy-induced apoptosis [43,44,45]. Furthermore, Hsps increase tumor invasion, angiogenesis, metastasis and stabilize and maturate oncogenic client proteins [46] that are involved in a number of signaling pathways such as the MAP kinase and NF-κB pathway [47,48,49]. Therefore, strategies silencing chaperones such as Hsp27 and Hsp70 either alone or in combination have been shown to enhance cancer cell death induced by Hsp90 inhibitors, and upon temozolomide and quercetin treatment of glioblastoma *in vitro* [50,51,52,53]. Alterations of post-translational expression of HSP70 by the stemness marker nestin has been shown to regulate tumor growth and invasiveness in glioblastoma via cyclin D1 and therefore an inhibition of both HSP70 and nestin might improve outcome of glioblastoma patients [54]. Another aspect for treating glioblastoma is the targeting of prion HSP90/70-organizing protein (HOP) complexes which have been found to be overexpressed in glioblastoma cells in mouse models [55,56].

Brown [57,58] could demonstrate that overexpression of members of the HSP70 family which can be induced either by celastrol or simply by heat shock can rescue normal mammalian brain tissue from ischemic injury and can result in a delayed progression of amyotropic lateral sclerosis (ALS) in mice. It appears that HSP70 derived from glial cells forms complexes with Hsp40 in synapse-rich areas of the brain which as a result can refold denatured proteins and stabilize centrioles which might be stress-sensitive especially in post-mitotic human neurons [59]. Furthermore, with respect to tumors it has been shown that HSPs with molecular weights of 70 and 90 kDa have been found to act as danger signals that can stimulate anti-cancer immune responses either by themselves or in combination with immunogenic peptides which are chaperoned by them [60,61,62]. Our group could demonstrate that Hsp70 is frequently presented on the cell surface of a multitude of different tumor entities [63,64,65] including glioblastoma (Ms in preparation). Membrane-bound Hsp70 was identified as a target for C-type lectin receptor-positive NK cells [66] that had been pre-stimulated with Hsp70 protein or an Hsp70 peptide in combination with the pro-inflammatory cytokine IL-2 [63,67,68,69]. Radiation, chemotherapy, hyperthermia and hypoxia are able to stimulate the synthesis of members of the HSP70 family including the major stress-inducible Hsp70 (HSPA1A) in the cytosol, increase the plasma membrane expression [70,71,72] and the release of Hsp70 in lipid vesicles [73]. The group of Capelli *et al*. [74] demonstrated a translocation of Hsp70 and HMGB1 (High-Mobility-Group-Protein B1) from the cytosol into the extracellular environment after an increased dose of irradiation. However, it remains to be determined which dose and which order of treatment is most effective to release danger signals from tumor cells. Elevated mRNA transcript levels of HSP70 [75] and elevated serum Hsp70 levels [76] have potential as prognostic biomarkers for glioblastoma and might help to monitor clinical outcome in the future.

## 4. Dose Escalation: Particle Radiotherapy for the Treatment of Gliomas—Promise or Not?

For GBM, a clear dose-response relationship is known. Early preclinical and clinical data have shown this, and studies have established the concept of “60 Gy” for GBM weighing local control *vs.* the risk of side effects [9]. Several approaches have been undertaken to make use of this dose-response-relationship, trying to increase the dose to the tumor to increase outcome. In this context the questions of dose-volume-effect must be kept in mind, and novel tools of target volume identification (MR-spectroscopy, PET or other) might be useful in this context. In the past studies of dose escalation were associated with an increase in toxicity, prototypely a study on proton therapy by Fitzek *et al.* [13], however, today, improvements in technique and target volume definition open new potentials in this direction. Improvement in radiation oncology, the implementation of high-precision techniques such as stereotactic radiotherapy or intensity modulated radiotherapy (IMRT), enable the radiation oncologist to deliver local dose escalation to defined subvolumes. Within a prospective phase III trial local dose escalation is currently addressed based on MR-spectroscopy; local dose escalation using a simultaneous integrated boost technique to a total dose of 72 Gy to the spectroscopic active region is evaluated (SPECTRO-GLIO-trial). Inclusion criteria are unifocal GBM with an indication for combined chemoradiation with TMZ. Not only advanced photon methods are used for dose escalation, but novel radiation qualities might have an additional impact, such as particle therapy.

The physical and biological benefit of particle beams has lead to early clinical applications in the 1950s and 60s for tumor treatments [77]. In Japan, a focus was set on carbon ion therapy, and at HIMAC (Heavy Ion Medical Accelerator, Chiba, Japan) patient treatments started in the mid-nineties. A further center using carbon ions started in 1997 in Darmstadt, Germany. Proton therapy has started initially in Loma Linda, CA, USA, being the first hospital-based proton facility, which is still treating cancer patients today.

Proton and heavy ion irradiation offer distinct physical characteristics contributing to an overall improved risk-benefit-profile in radiotherapy. The high precision of the particle beam is based on the physical properties, with an inverted dose profile. While in the entry channel, only low doses are deposited, a high local dose deposition in the so called Bragg Peak can be directed by the particle energy precisely into tumor tissue [78,79]. This increased therapeutic ratio may also permit dose escalation to the tumor and therefore leads to an increased tumor control.

Proton beams can be deposited in precise areas with minimal lateral scattering; this ensures that only little radiation is delivered to healthy normal tissue surrounding the tumor [80,81]. This advantage makes proton radiotherapy to a preferred option for treating central nervous malignancies including glioblastoma. Proton radiation induces at least 10% higher relative biological effectiveness (RBE) in most types of cells and tissues when compared to conventional photon therapy. A recent study indicated that proton radiation induces more apoptosis than photon radiation in various cancer cell types [82]. Previous results show that proton therapy provides better local control in meningioma [83]. Several clinical trials with proton radiotherapy have been conducted for glioblastoma patients. Fitzek *et al.* conducted a phase II study with 23 glioblastoma patients which were treated with 90 Gy of X-rays or protons using hyper-fractionated radiotherapy [13]. The median survival time was extended to 20 months, likely as a result of central control. The study of Mizumoto and coworkers recently reported the results of a phase I/II trial evaluating the role of a proton boost therapy with dose escalation up to 96.6 Gy for glioblastoma patients [84]. The group also demonstrated excellent median and overall survival rates compared to historical controls. The study represents one of the few available examining clinical dose escalations with proton therapy in the CNS. The median survival time was extended to 21.6 months, which is one of the most favorable results reported so far. In another study Mizumoto and coworkers evaluated glioblastoma survivors after postoperative hyper-fractionated concomitant boost X-ray radiation therapy and proton beam therapy [85]. The results demonstrated that high-dose proton beam therapy could effectively control glioblastoma if the treatment area completely covers tumor infiltration. Currently, a randomized phase II trial is comparing hypofractionated dose-escalation with IMRT or protons to conventional 60 Gy radiotherapy in patients with newly diagnosed supratentorial GBM (NRG-BN001)—the primary endpoint is overall survival. This concept will address—once again—the concept of dose escalation, allowing the application of proton therapy, when available.

High linear energy transfer (LET) radiotherapy using carbon ions comprises an increased radiobiological efficacy, especially with respect to the Bragg peak [80]. Therefore, heavy ion irradiations have a higher RBE. The RBE of heavy ions is optimal at the Bragg Peak, but the Bragg Peak is also tissue-dependent. For glioblastoma cell lines the RBE has been shown to be between 3 and 5 for carbon ions [81]. High-LET heavy ions also have additional biological advantages compared to protons or X-rays, decreased oxygen enhancement ratio, reduced cell cycle-dependency and the potential of metastases suppression [86,87,88]. Because of its physical and biological advantages, carbon ion radiotherapy is a promising modality in the treatment of patients with glioblastoma. The potentially beneficial effect of carbon ion radiotherapy in glioblastoma patients has been evaluated in the preclinical as well as clinical setting within prospective trials [89,90]. Combs *et al.* investigated the cytotoxic effect of carbon irradiation on glioblastoma cell lines in combination with temozolomide [90]. The authors demonstrated that carbon ion irradiation is significantly more effective for glioblastoma cell lines compared to photon irradiation. First clinical results from Chiba, Japan, showed low toxicity but good efficacy of carbon ion radiotherapy in patients with glioblastoma in a phase II trial [91]. The treatment consisted of 25 fractions and a total dose of 50 Gy X-ray radiotherapy and carbon ion radiotherapy with the doses increased from 16.8 to 24.8 GyE in 10% incremental steps. The late reactions included four cases of grade 2 brain toxicity among 48 cases. However, no grade 3 acute toxicity in the brain was found. The median survival time of glioblastoma patients was 17 months and the median progression-free survival 7 months for the low-dose group, 19 months for the middle-dose group, and 26 months for the high-dose group. In another study, Combs and co-workers compared retrospectively the outcome after photon radiotherapy alone, radio-chemotherapy with temozolomide and carbon ion radiotherapy in patients with high-grade gliomas [92]. The median progression-free survival for patients was 5 months in the radiotherapy group, 6 months in the radiotherapy plus temozolomide group, and 8 months in the carbon ion group. The results showed a significant difference between the radio-chemotherapy group and the carbon ion group. The study demonstrated a potential benefit of carbon ions in patients with high-grade gliomas, however it has clear limitations in terms of the retrospective nature of the analysis and the pooled data structure from two institutions.

## 5. Vaccination Strategies for Glioblastoma

The goal of any cancer vaccine is the induction of anti-tumor immune responses or the expansion of preexisting immunity against tumor antigens which are effective in mediating tumor regression [93]. Antigens expressed within tumor cells can be classified into two general categories: tumor-associated antigens (TAAs) and tumor-specific antigens (TSAs). TAAs may serve as immunogenic targets for the immune system [94]. Several TAAs have been evaluated in therapeutic vaccine trials in patients with primary or recurrent glioblastoma. In the study of Freitas *et al.* an extensive expression analysis of cancer/testis antigens (CTAs) within glioblastoma was conducted using 153 CTA genes. Using RT-PCR detection within normal brain as exclusion for further consideration, these investigators identified four genes (ACTL8, CTCFL, OIP5, and XAGE3) as candidate CTAs uniquely expressed within glioblastomas [95].

There is a clear advantage by making use of whole tumor lysate as an antigen source as it serves as a widespread and patient-specific bank of potential immunologic targets [96]. Tumor lysate vaccines are generated by culturing resected tumors and isolating the surface proteins. First results using this approach were published in 2000 and in 2005 showing no major adverse events with the vaccine, however only a non-significant increase in survival [97,98]. Several other studies were conducted and showed that certain glioblastoma patients are more responsive than others [99,100]. The results indicated that patients of the mesenchymal glioblastoma subgroup showed better survival and improved T cell infiltration of the tumor than patients of other glioblastoma subgroups [101].

Epidermal growth factor receptor (EGFR) signaling has an important role in many cancers, as proliferation, differentiation and migration is mainly controlled by growth factors and their receptors [102]. Epidermal growth factor receptor variant type III (EGFRvIII) is a mutation that is heterogeneously expressed in 24%–67% of primary glioblastoma [103]. The mutated protein is not found in any normal tissues. Therefore, several groups have researched ways to target EGFRvIII in glioblastoma. Focussing on EGFRvIII-expressing glioblastoma, a prospective phase II trial was performed to evaluate immunogenicity, outcome in terms of progression-free survival (PFS) and overall survival (OS) after a peptide-based vaccine (PEPvIII) [104]. The study detected no symptomatic autoimmune reactions. The 6-months PFS rate after vaccination was 67% and after diagnosis 94%. The median overall survival was 26.0 months (95% CI, 21.0 to 47.7 months). Several studies of vaccines targeting EGFRvIII in glioblastoma patients have been completed and more are underway [104,105,106]. In summary, the different EGFRvIII vaccine trials demonstrated the safety and feasibility of these vaccines [96]. However, it has been previously shown that targeting the EGFR receptor can lead to selection pressure for somatic mutations and may lead to resistance to EGFR inhibitors, which could be also a problem in immune therapy using vaccines [107].

Another development in glioblastoma immunotherapy is the vaccination with dendritic cells (DCs). After stimulation, DCs can migrate to draining lymph nodes where they induce immune responses [108]. One of these dendritic-cell-based vaccines is called DCVax-L^®^. It is a vaccine in which autologous DCs are pulsed with a lysate derived from the patient’s own resected tumor. In another trial immunogenic lysates plus dendritic cells from peripheral blood cells stimulated with granulocyte-macrophage colony-stimulating factor and Interleukin-4 were evaluated in a phase I clinical trial with 23 patients after surgical resection of GBM [101]. The study demonstrated that DC vaccinations are safe with no dose-limiting toxicity. The median overall survival time was 31.4 months, with a 1-, 2-, and 3-year survival rate of 91%, 55%, and 47%, respectively [101]. In another study, Yamanaka and colleagues showed an enhanced overall survival of 480 days in those patients with grade 4 glioma compared to 400 days in the control group [109]. Interestingly, the best survival was achieved in patients receiving both intra-dermal and intra-tumoral vaccination, which could be attributed to activation of both central and peripheral immune responses.

Tumor-derived heat-shock proteins (HSPs) can also be used for vaccination in glioblastoma patients. HSPs are known to bind receptors expressed by DCs resulting in CD4 and CD8 T-cell responses. Crane and coworkers investigated HSP96 for use as a cancer vaccine [62]. Twelve patients with recurrent glioblastoma were treated with tumor-derived HSP peptide complex consisting of HSP96 and a broad array of tumor-associated antigenic peptides. The study showed that immune responders had a median survival of 47 weeks after surgery and vaccination, compared to 16 weeks for the single non-responders.

## 6. Innovative Radiotherapy (iRT)

iRT includes modern radiation and makes use of tumor and environmental properties such as the immune system, molecular markers or oxygenation status of the tumor and its microenvironment. The current manuscript sheds light on 4 main topics in this context, taking note of the limitation that there are more potential points of action.

Novel radiation techniques enable local dose escalation, making use of the known dose-response relationship of GBM. However, integration of improved imaging is an essential asset compared to older dose escalation trials, since subvolumes at high risk can be identified. These regions can be dose-escalated locally, while minimizing the risk for treatment-related side effects, which were a major issue in the past with dose escalation. The identification of high risk regions, let them be hypoxic, extensively proliferative, tightly interconnected or other, might be the key combination with modern high-precision radiotherapy. Dose escalation strategies to subvolumes of the tumor representing high-risk regions such as within simultaneous integrated boost concepts or with application of particle therapy are currently under investigation. Here, combination of those technologies together with treatment targeting the tumor and its micromilieu, or approaches making use of the immune system. Combination of immune-modulating agents and radiotherapy might have the potential to overcome the overall treatment resistance in GBM by making use of the tumor’s own immunogenic properties; promising combinations of substances such as ipilimumab or nivolumab are currently under investigation (e.g., CheckMate 143-Study). To date, several studies targeting molecular assets of the tumor have often not been successful; explanations might be the type of patient recruitment not focusing on patient subgroups with most benefit from the respective approach, or iRT makes use of not only novel technical developments or integration of imaging, but driving treatment by true personalized approaches understanding the mechanisms of treatment resistance. The unique growth pattern of GBM with tumor cells in astrocytomas having ultra-long membrane protrusions as well as distinct tumor microtubes. These are used as routes for brain invasion, proliferation, and to interconnect over long distances. Thus, a network based on multicellular communication through microtube-associated gap junctions can be used also for damage repair. In summary, astrocytomas can develop functional multicellular network structures. Thus, disruption of these microtubuli and the resulting network might be a novel approach to overcome treatment resistance [110]. The concept of TGF-β signaling and the important role in tumor development and growth is revisited on several occasions, and a potentially important role in GBM biology has been shown [111,112,113,114,115]. Thus, novel concepts of targeting TGF-β in combination with radiotherapy and/or chemotherapy are worth investigation within the context of iRT.

Ideally, individualized treatments close the loop from diagnosis, surgery and adjuvant treatment precisely selecting patients who benefit most from each of the key components of treatment. The heterogeneity of brain tumors with respect to immunogenic properties, molecular characteristics as well as clinical parameters requires strong interdisciplinary work and reliable neuropathological classifications.

**Figure 1:**
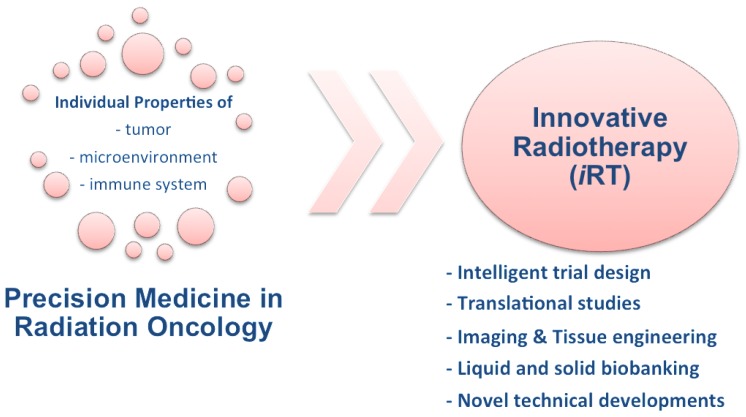
Precision medicine in the context of Innovative Radiotherapy (iRT).

Integration of several of these mechanisms, or perhaps one essential checkpoint, requires extensive preclinical research, and subsequently prospective randomized clinical trials. Regarding the overall low incidence and the recruiting and follow-up times, intelligent trial designs are mandatory to acquire solid results within acceptable time frames. Thus, the road for iRT is summarized in Figure 1 and serves as a roadmap for the next research-oriented years to come.
